# Shot-noise-limited X-ray powder diffraction from organic crystals with laser-driven femtosecond X-ray source

**DOI:** 10.1107/S1600576726009040

**Published:** 2026-09-15

**Authors:** Iuliia Baranova, Yelyzaveta Pulnova, Roya Garayeva, Yadu Ram Panthi, Gabriel Žoldák, Jaroslav Nejdl, Borislav Angelov, Filip Leonarski, Jakob Andreasson, Krishna Khakurel

**Affiliations:** a ELI Beamlines Facility, Extreme Light Infrastructure ERIC, Za Radnicí 835, 252 41Dolní Břežany, Czechia; b Faculty of Mathematics and Physics Charles University 121 16 Prague Czechia; c Czech Technical University in Prague FNSPE Břehová 78/7 115 19 Prague Czechia; d Institute of Macromolecular Chemistry CAS Heyrovského nám. 2 16206 Prague 6 Czechia; e Faculty of Science, Pavol Jozef Šafárik University in Košice, Park Angelinum 19, 040 01Košice, Slovakia; f Paul Scherrer Institute Forschungsstrasse 111 Villigen5232 Switzerland; SLAC National Accelerator Laboratory, Menlo Park, USA

**Keywords:** laser-driven X-ray sources, femtosecond X-rays, powder diffraction, structural dynamics, fast detectors

## Abstract

Laser-driven X-ray sources are increasingly used to investigate the transient behaviour of optically excited materials. While their application to thin films and powders of radiation-hard samples has been extensively explored, their use for radiation-sensitive materials such as organic and protein crystals remains limited. A key challenge is the low signal-to-noise (S/N) ratio, caused by the inherently low flux of these sources and additional non-Poissonian noise arising from temporal fluctuations. Improving noise suppression and enhancing the S/N ratio are therefore critical steps toward extending the applicability of these sources to more demanding systems. We demonstrate shot-noise-limited X-ray powder diffraction from 4-phenylazophenol, representing an organic crystal, using a laser-driven plasma X-ray source. These results highlight the potential of such sources for future studies of transient structural dynamics in photo-excited organic crystals.

## Abstract 2

This communication highlights the feasibility of recording shot-noise-limited X-ray powder diffraction from organic crystals using a laser-driven X-ray source.

## 1. Introduction

Tracking photo-excited dynamics in solids, organic materials and macromolecules is an active area of research. A wide variety of systems have been studied using time-resolved optical spectroscopy over the past three decades (Oliver, 2018 ▸). While these studies have established a rich understanding of photophysical processes and their associated timescales, they do not provide direct atomistic insight into the structural dynamics. This limitation has been addressed through the development of transient structural methods based on short-wavelength radiation such as X-rays (Marangos, 2019 ▸).

Femtosecond X-ray pulses can be generated using accelerator-based sources such as free-electron lasers, synchrotron slicing techniques or laser-driven X-ray sources (Schoenlein *et al.*, 2019 ▸). Among these approaches, laser-driven sources can be realized in compact, laboratory-scale setups at relatively low cost. Furthermore, using a fraction of the same driving laser as the pump beam enables inherently jitter-free time-resolved measurements. Such sources have been used to probe dynamics in a range of systems (Afshari *et al.*, 2020 ▸; Khakurel *et al.*, 2025 ▸; Gonzalez-Vallejo *et al.*, 2025 ▸). However, their moderate flux limits the range of accessible samples. While efforts to develop brighter tabletop X-ray sources are ongoing (Shen *et al.*, 2024 ▸; Koç *et al.*, 2021 ▸), improving the signal-to-noise ratio is equally important to expand the range of measurable systems.

X-ray powder diffraction (PXRD) is a widely used structural characterization technique in which polycrystalline samples are illuminated with X-rays and the resulting Debye–Scherrer rings are recorded using one- or two-dimensional detectors. Time-resolved powder diffraction with laser-driven X-ray sources has been extensively applied to study photoinduced dynamics in crystalline solids and thin films (Zamponi *et al.*, 2010 ▸; Freyer *et al.*, 2013 ▸; Hauf *et al.*, 2019 ▸), and more recently in organic thin films (Gonzalez-Vallejo *et al.*, 2025 ▸). We have previously discussed the potential of time-resolved PXRD for macromolecules using such sources (Khakurel *et al.*, 2024*b* ▸). However, broader application requires improved detection of weak difference signals.

Unlike accelerator-based X-ray sources, where photon statistics are dominated by shot noise, laser-driven plasma X-ray sources (PXSs) based on tape targets exhibit significant temporal fluctuations in photon yield. Such sources are best described as an intermediate case between pure Poisson light and a chaotic light which follow a Bose distribution (Holtz *et al.*, 2017 ▸):
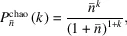
where 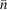 is the average photon flux per pulse and *k* is the number of detected photons per pulse. This deviates from Poisson statistics due to enhanced intensity fluctuations. Reducing temporal fluctuations can therefore enable shot-noise-limited performance.

Here, we present shot-noise-limited PXRD from a laser-driven source, enabled by shot-to-shot detection of both diffracted and transmitted beams using the Jungfrau detector (Leonarski *et al.*, 2018 ▸) and the Jungfraujoch data acquisition system (Leonarski *et al.*, 2023 ▸). While near shot-noise-limited diffraction has previously been demonstrated for strongly scattering samples (Holtz *et al.*, 2017 ▸), it has not been shown for kHz repetition rate PXSs, where temporal fluctuations are expected to be more pronounced. Only recently have similar capabilities been demonstrated in time-resolved electron diffraction (Claude *et al.*, 2025 ▸). Compared with electron diffraction setups, copper-tape-based PXSs exhibit significantly higher fluctuations. Extending shot-noise-limited detection to such systems is therefore of broad relevance. We also briefly discuss the benefits and prospects of integrating a continuous X-ray source within the same setup.

## 2. Experiments

**Figure 1:**
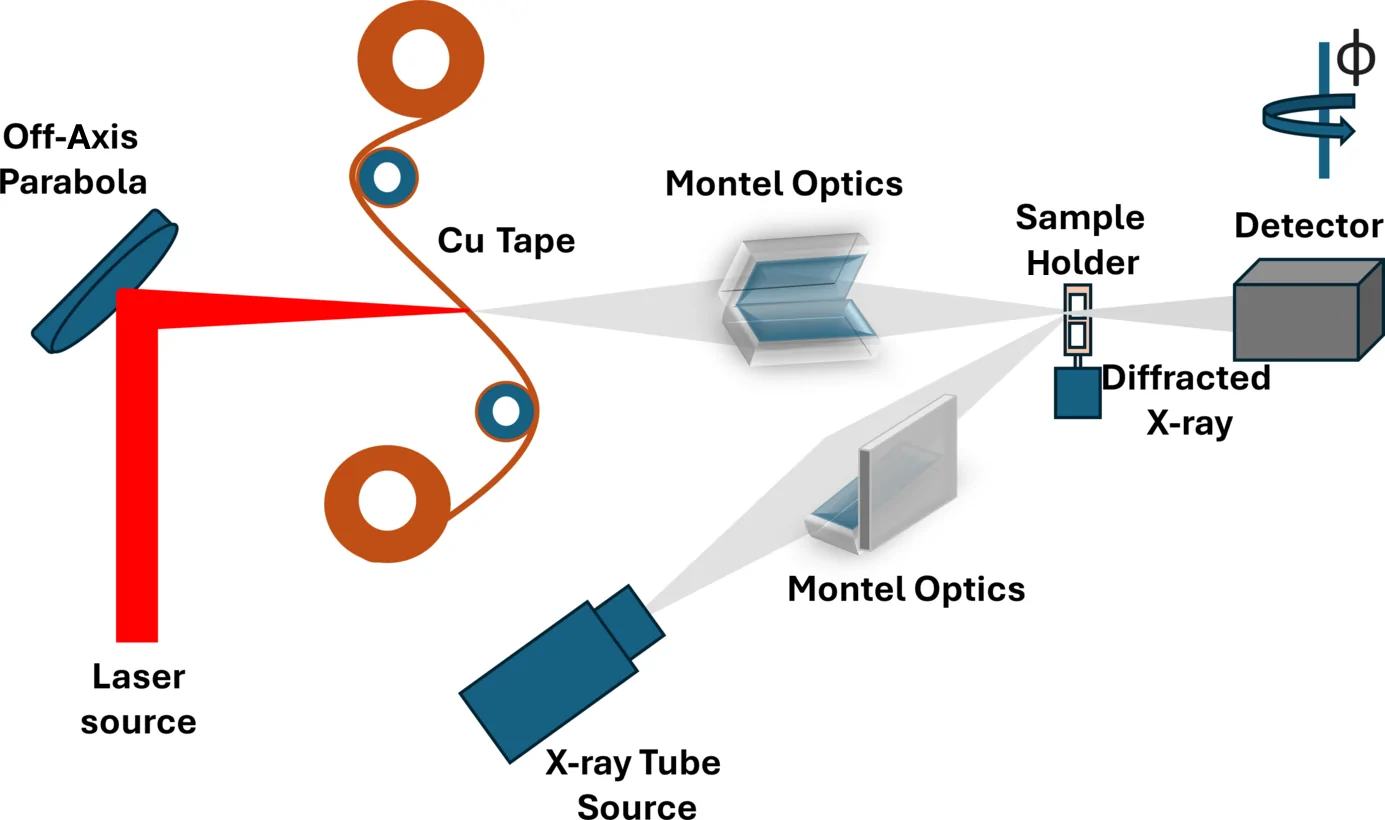
A schematic of the experimental setup at the TREX end station in the E1 experimental hall at ELI beamlines.

The experiments were conducted at the TREX end station at ELI Beamlines (Fig. 1 ▸). The setup comprises a femtosecond laser-driven X-ray source complemented by a continuous X-ray source. The presence of a continuous source enables optimization of sample conditions prior to femtosecond measurements and facilitates estimation of the total acquisition time required to obtain meaningful signals. Additional advantages of this configuration are discussed in the *Conclusion and outlook* section[Sec sec5].

The femtosecond X-ray source is driven by a 1 kHz laser system (Legend Duo Elite, Coherent Inc.) with a pulse energy of ∼12 mJ, central wavelength of ∼800 nm and pulse duration <40 fs. The laser is focused to a spot of ∼10 µm using an off-axis parabola onto a ∼20 µm-thick copper tape target, which is refreshed after each shot. The generated X-rays are collected and focused using multilayer Montel optics (AXO Dresden GmbH), which also act as a spectral filter. The focal spot at the sample plane is ∼150 µm (FWHM). A detailed description of the source and the TREX end station are given by Pulnova *et al.* (2025 ▸).

The sample is mounted on a five-axis stage in a custom holder. For this study, 4-phenylazophenol was used as the model system. The detector was positioned at a nominal distance of 66.7 mm from the sample, calibrated using LaB_6_ powder diffraction. This geometry provides an accessible *q* range up to 2.0 Å^−1^ at the detector centre and up to 4.0 Å^−1^ at the detector edge.

Data acquisition employed a PSI Jungfrau 1.5M detector (Leonarski *et al.*, 2020 ▸), a charge-integrating hybrid pixel detector with dynamic gain switching. Each pixel (75 × 75 µm^2^) can operate in multiple gain stages, enabling both single-photon sensitivity and high-intensity measurements without saturation. The detector was calibrated following standard PSI procedures, including pedestal measurements, gain calibration using fluorescence and pixel-wise threshold adjustment. Dead pixels and module gaps were identified and masked.

Precise shot-to-shot correlation between X-ray generation and detection is critical for this method. The detector is triggered by TTL pulses provided by the laser and synchronized with the X-ray-producing pulses through a programmable delay of 995 µs. Given the laser repetition period of 1 ms, this ensures that the detector’s 10 µs acquisition window captures the X-ray pulse generated by the subsequent laser shot (*N* + 1) after receiving trigger *N*.

To verify the synchronization accuracy, we monitored the transmitted beam intensity while intentionally varying the delay in 1 µs steps. The intensity exhibited a sharp maximum at a delay of 995 µs, with an FWHM < 2 µs, confirming appropriate timing alignment.

The timing jitter between the laser and detector is dominated by the laser system’s pulse-to-pulse jitter, typically <100 ps RMS. This is negligible compared with the 10 µs acquisition window.

**Figure 2:**
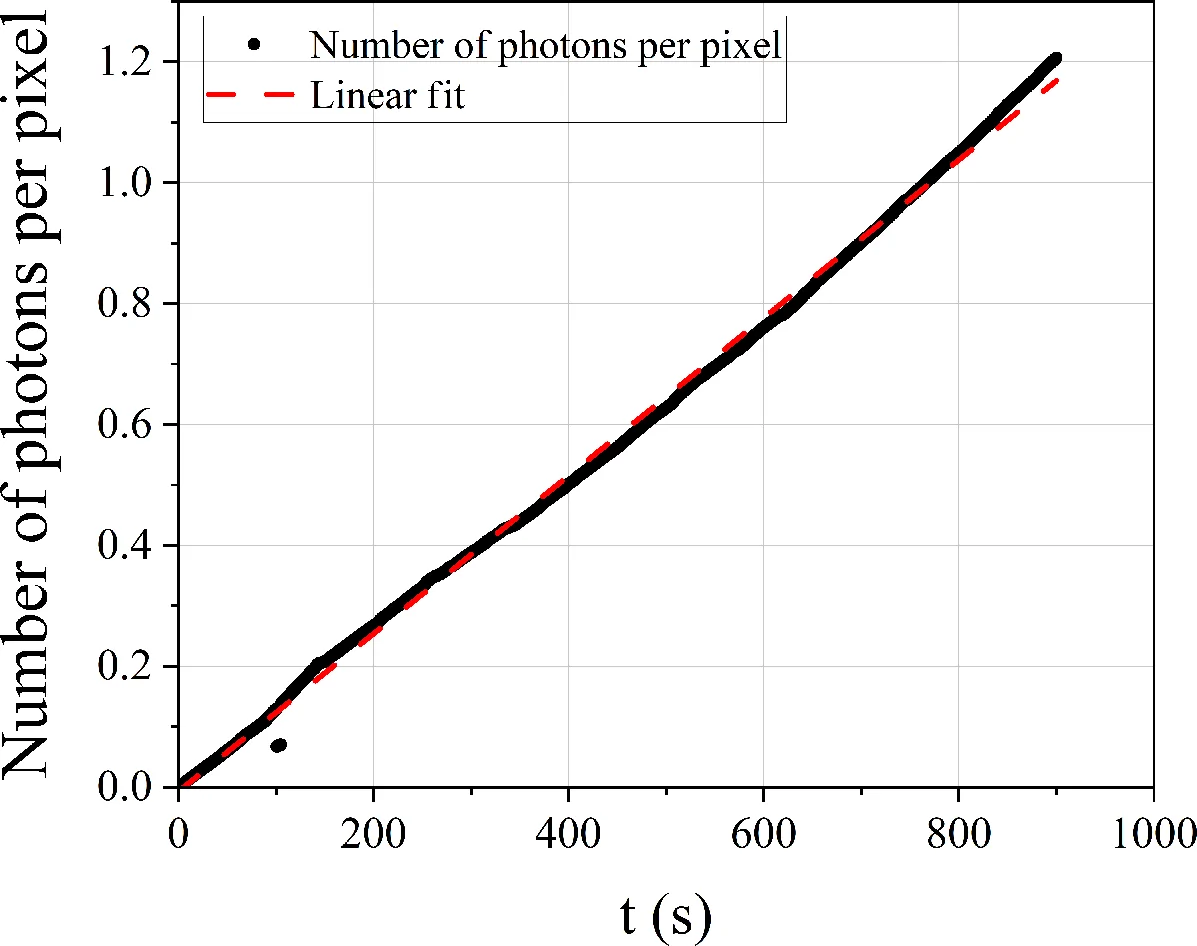
The background recorded by the detector over a time of 15 min in the absence of X-ray radiation. A linear fit of the data is shown by the dotted line.

The background recorded by the detector in shot-to-shot mode under the experimental conditions is shown in Fig. 2 ▸. The contribution from cosmic background amounts, on average, to ∼1 photon pixel^−1^ over 15 min of data acquisition. Such low backgrounds are critical while measuring samples with smaller atomic numbers, such as the organic molecule used for this study.

Data acquisition was performed using the Jungfraujoch system (Leonarski *et al.*, 2023 ▸). The acquisition server is a Dell PowerEdge R760 equipped with two Intel Xeon 6448Y CPUs, 768 MB of RAM and an AMD U55C FPGA card. The FPGA receives image data from the detector via 10 Gbit s^−1^ fibre-optic links, converts the raw readout into 8 keV photon counts and performs basic image processing. The CPU component of Jungfraujoch applies image compression using the Bitshuffle/LZ4 algorithm (Masui *et al.*, 2015 ▸) and writes NeXus-compatible HDF5 files (Bernstein *et al.*, 2020 ▸). The detector is controlled via a web interface written in TypeScript and React as part of the Jungfraujoch framework.

### 2.1. Sample preparation

**Figure 3:**
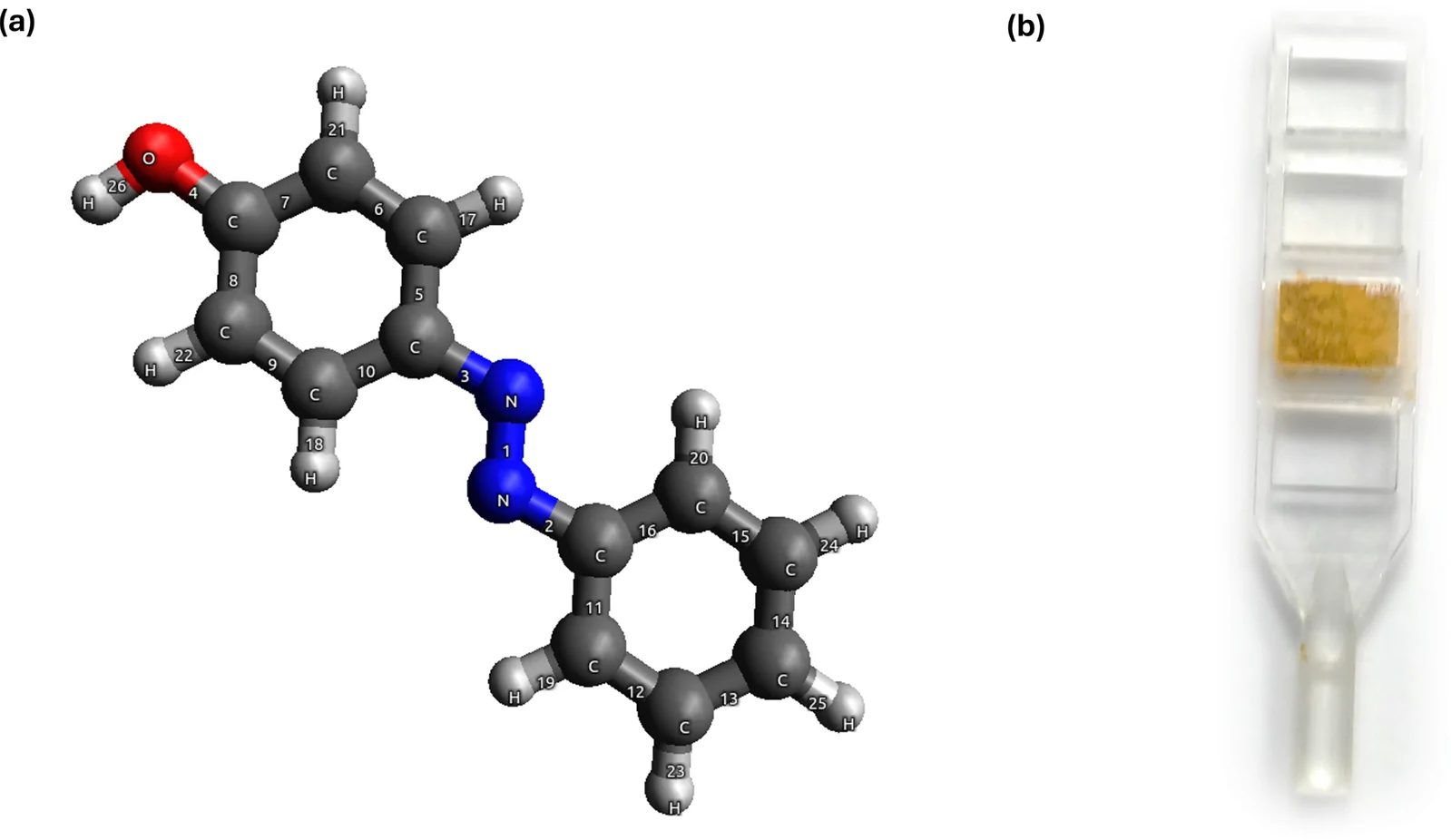
(*a*) The chemical structure of the 4-phenylazophenol used for the experiment. (*b*) The 3D-printed sample holder for X-ray powder diffraction with 4-phenylazophenol prepared for the experiment.

The sample, 4-phenylazophenol (CAS number: 1689-82-3), was purchased from Sigma–Aldrich and was used directly from the commercially outsourced bottle without any further purification/processing. No further crystallization optimization was performed for the current experiment. A schematic of the structure of the molecule used in the current study is shown in Fig. 3 ▸(*a*). The yellow powder of phenol azobenzene is sandwiched in the custom-designed holder as shown in Fig. 3 ▸(*b*). The frame of the sample holder is 3D printed with a central aperture of 3 mm diameter and depth of 0.5 mm. The holder is suitable for both powder and single crystals. The holder was sealed on both sides by Cyclic Olefin Copolymer (COC) film. The negligible background from COC film has been confirmed in previous X-ray diffraction experiments (Khakurel *et al.*, 2024*a* ▸).

## 3. Data collection and analysis

The results presented here include data collected from both the continuous X-ray source and the PXS. Data collection was performed in two modes:

(1) Integrated mode: frames are summed internally over a defined period (typically 1 s), saving only the integrated image. A 15 min measurement at 1 kHz produces 900 integrated frames.

(2) Shot-to-shot mode: each detector frame corresponding to a single laser pulse is recorded individually, resulting in ∼9 × 10^5^ frames for a 15 min acquisition.

The estimation of the diffraction signal from PXS was done using a conventional tube source. The two-dimensional PXRD pattern was collected using a conventional X-ray tube source providing X-rays at copper *K*α (1.54 Å) with an acquisition time of ∼15 min and flux ∼2.36 times higher than that provided by the laser-driven Cu *K*α source. The two-dimensional PXRD pattern is then azimuthally averaged and reduced to a 1D diffraction profile. The blind region of the detector and all dead pixels were masked out. An example of such a mask is provided in Supplementary Fig. 2. For each azimuthally averaged diffraction pattern, a baseline was computed by fitting a third-order polynomial which was observed to adequately model the baseline. We also tried lower-order polynomials, but from our experience the third-order polynomial provided the best estimates of the baseline. All the radial PXRD patterns shown in this article were obtained after subtracting the baseline, unless explained otherwise. An example of the raw two-dimensional PXRD from the continuous source and the PXS is shown in Supplementary Figs. 1(*a*) and 1(*b*), respectively.

To measure the powder diffraction pattern using the PXS, the sample was then rotated using the five-axis stage to align the sample in an orthogonal position with respect to the X-ray beam from PXS. The powder diffraction was collected from a position of the sample that was almost identical to that used for the PXRD measurement with the conventional tube source. The data were collected in both shot-to-shot mode and 15 min integrated mode for making the comparison. During the data collection the detector was run at a frequency of 1 kHz. For the integrated images, the summing of the images was done internally, and a single image frame was received as the output.

The spatially structured intensity gradient observed in the diffraction pattern recorded with the PXS [Supplementary Fig. 1(*b*)] was due to the portion of the direct beam which was not reflected by the Montel optics. The intense part of this structured intensity was masked during the analysis. The remaining parasitic scattering may contribute to deviations from ideal Poisson statistics. During the shot-to-shot analysis, all detector pixels with photon counts exceeding the median photon count of the transmitted beam were excluded. This procedure significantly reduced the influence of the parasitic scattering. Introduction of a collimator downstream of the Montel optics can get rid of such structured intensity gradients, which is planned as a future development of the beamline.

To quantify the benefits of shot-to-shot acquisition, a full dataset of ∼900 000 frames (15 min at 1 kHz) was recorded, each with a 10 µs acquisition window. Shot-to-shot acquisition allows the identification and removal of invalid frames (*e.g.* due to target instability) and enables correction of the beam position and flux fluctuations. The use of a two-dimensional detector ensures simultaneous collection of multiple Debye–Scherrer rings, providing richer structural information. Additionally, the Jungfrau detector allows simultaneous measurement of the transmitted beam, enabling normalization and monitoring of beam stability.

## 4. Results and discussion

**Figure 4:**
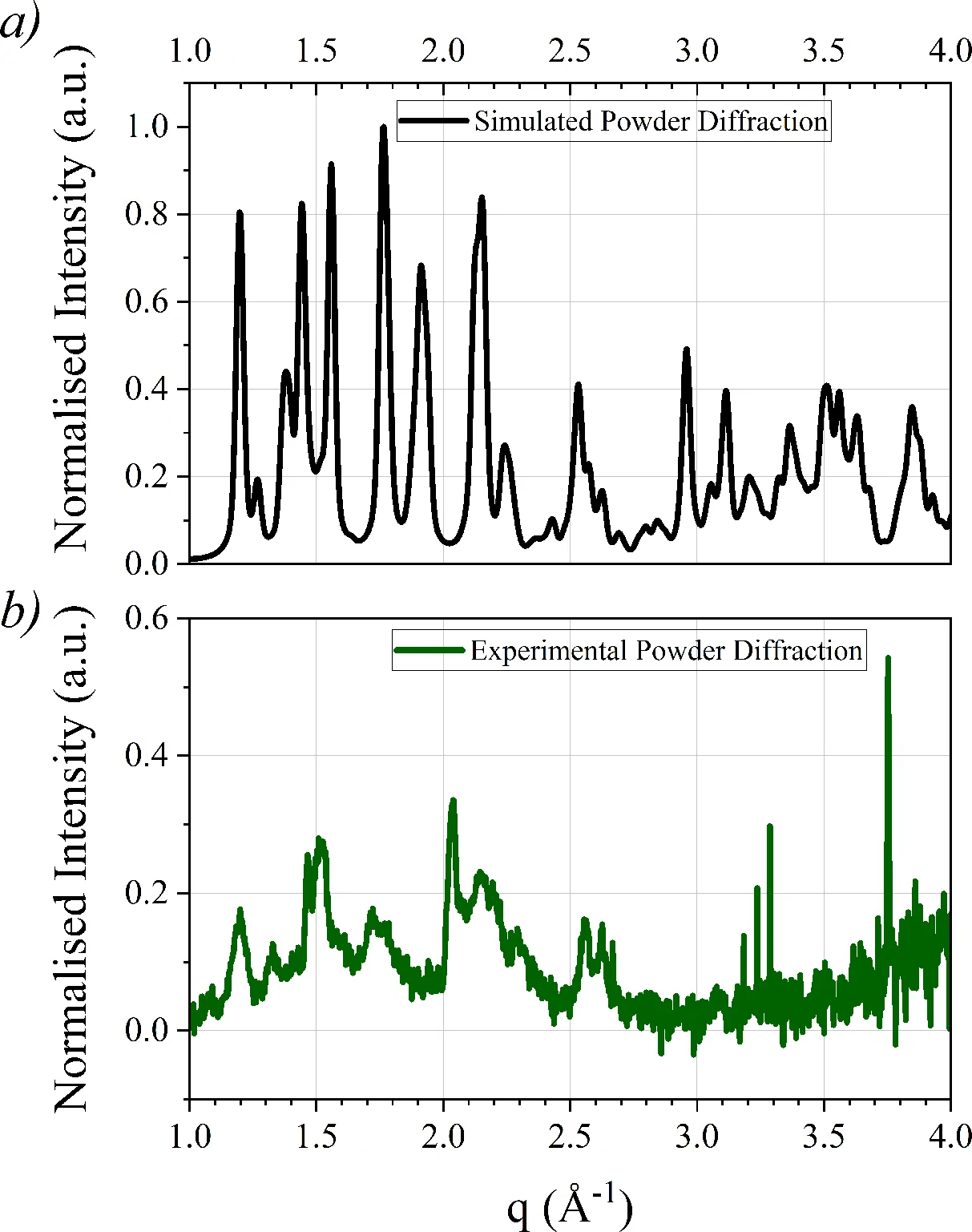
(*a*) The simulated PXRD profiles of 4-phenylazophenol and (*b*) the measured PXRD using the continuous Cu X-ray source with acquisition time of ∼15 min.

The azimuthally integrated one-dimensional PXRD profile derived from measurements with the continuous X-ray source is shown in Fig. 4 ▸(*b*). A simulated PXRD pattern based on a deposited CIF (Cambridge Crystallographic Data Centre ID: 1199907; Shamuratov* et al.*, 1991 ▸) is shown alongside the experimental data for comparison [Fig. 4 ▸(*a*)]. The simulation was performed using the *scikit-ued* package (René de Cotret, 2018 ▸).

**Figure 5:**
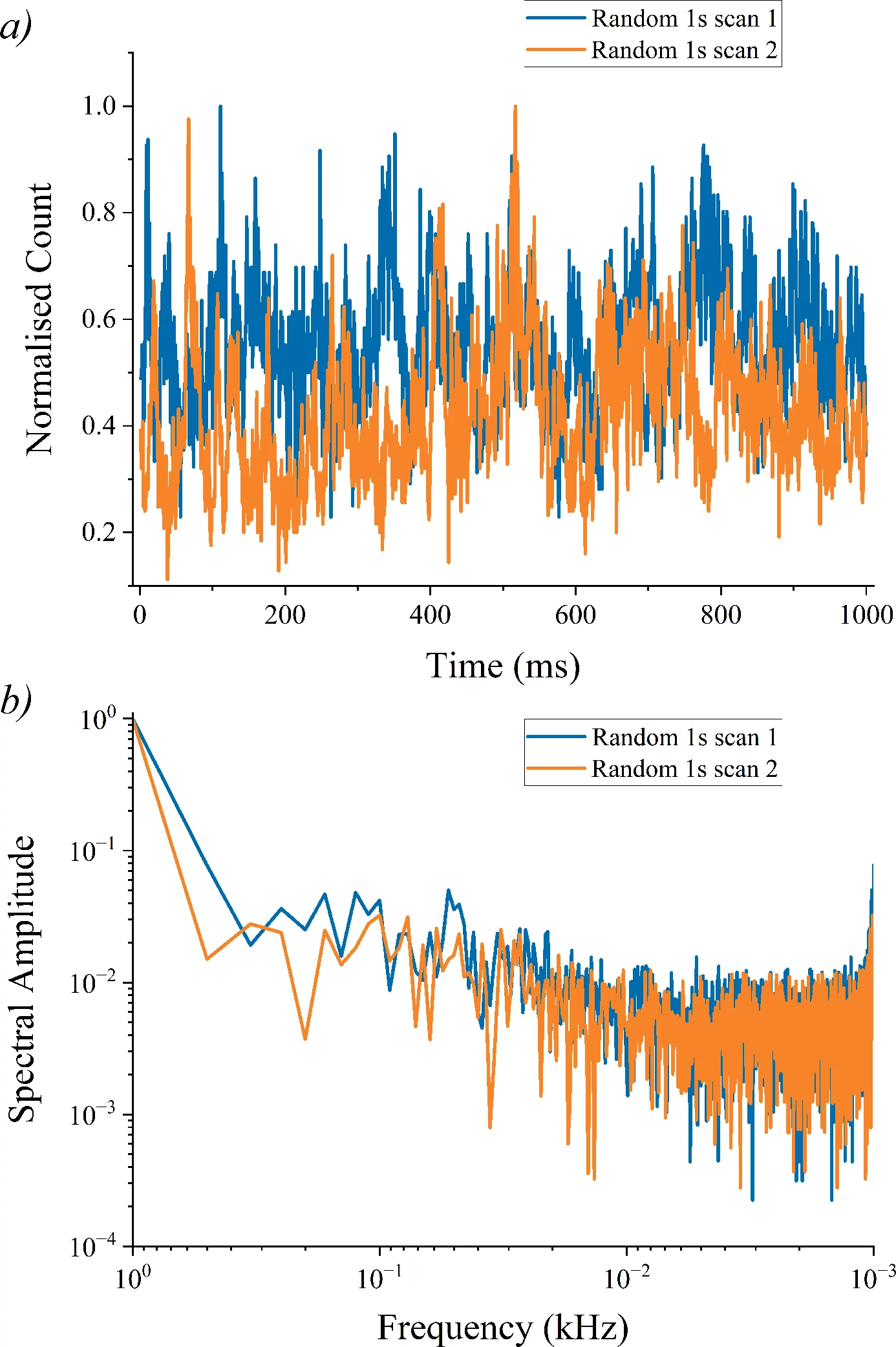
Integrated intensity of the transmitted beam measured with the plasma X-ray source for two random scans of 1 s in shot-to-shot mode. The Fourier transform of the plots in (*a*) is shown in (*b*).

The use of the Jungfrau detector provides the possibility of measuring a direct transmitted beam, which facilitates in monitoring the intensity of the transmitted X-ray beam and the centre of the X-ray beam. Fig. 5 ▸(*a*) shows the integrated intensity of the transmitted beam for two random scans of 1 s in shot-to-shot mode. The Fourier transform of the plots in Fig. 5 ▸(*a*) is shown in Fig. 5 ▸(*b*). The figure shows almost three orders of magnitude difference in the spectral amplitude between high frequency and low frequency. The position of the transmitted beam was also tracked along both *X* and *Y* directions (Supplementary Fig. 3). Shot-to-shot detection enables correction of beam-centre shifts, which can further improve the signal-to-noise ratio and reduce peak smearing. The root-mean-square deviation of the beam centre was 0.189 and 0.23 pixels (for a detector pixel size of 75 µm) in the *X* and *Y* directions, respectively. This corresponds to a small change in the detector geometry. Re-integration of the diffraction data showed that the resulting differences in the integrated dif­frac­tion profiles were negligible and did not affect the conclusions of the study. While this correction had limited impact for the weakly scattering sample studied here, it is expected to be beneficial for strongly scattering samples or brighter sources.

**Figure 6:**
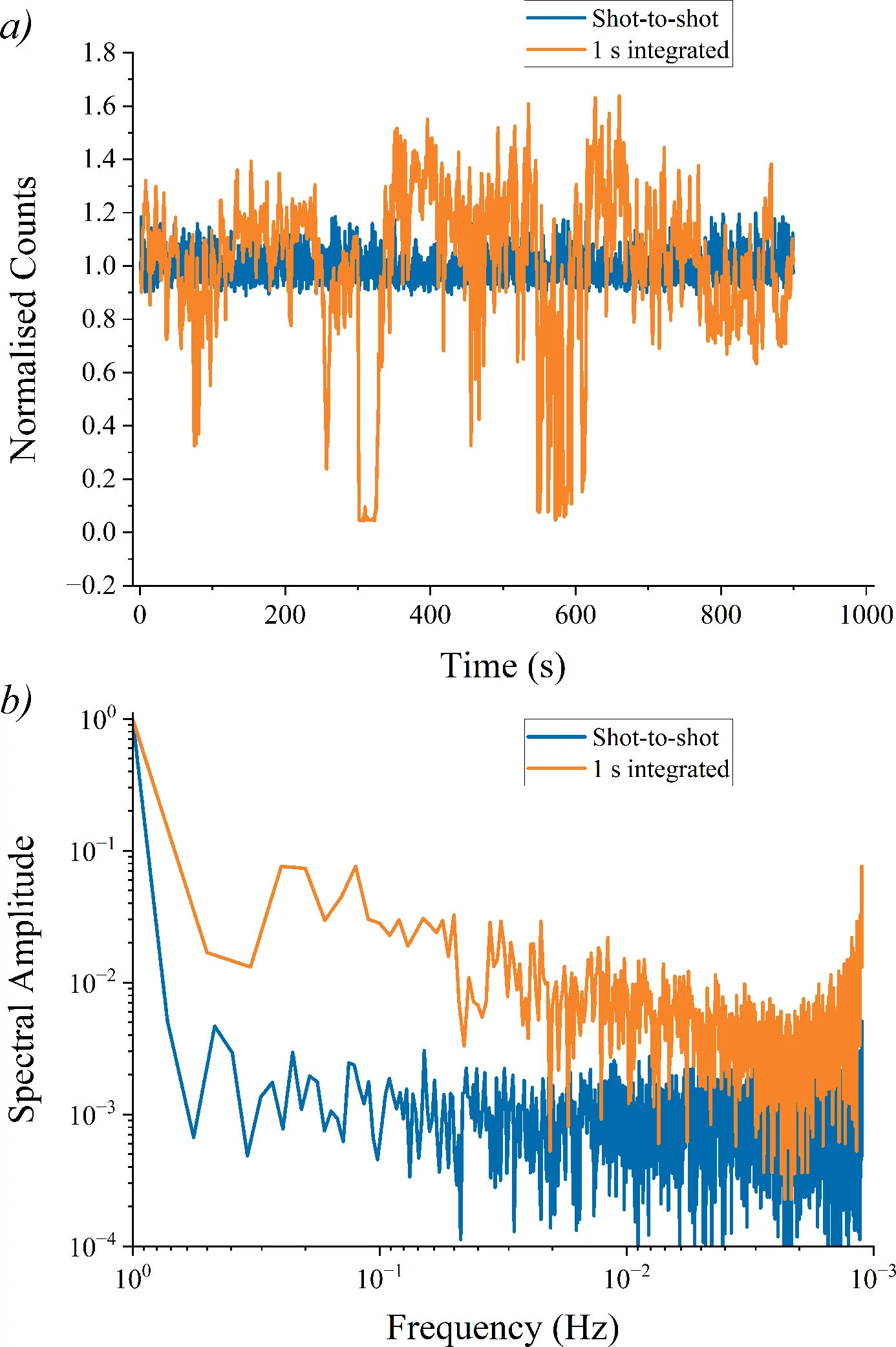
(*a*) The integrated diffraction intensity from the entire detector area from 4-phenylazophenol recorded over 15 min using the PXS and Jungfrau detector. (*b*) Fourier transform of the plots in (*a*).

Several strategies have previously been proposed to mitigate shot-to-shot fluctuations in laser-driven X-ray sources, often requiring additional instrumentation for normalization. In contrast, the Jungfrau detector enables direct measurement of the transmitted beam without saturation effects, eliminating the need for additional detectors. Shot-to-shot normalization using the transmitted beam significantly reduces noise. Fig. 6 ▸(*a*) shows a comparison between normalized shot-to-shot intensity and 1 s integrated data obtained using the PXS, while the corresponding Fourier spectra [Fig. 6 ▸(*b*)] illustrate differences in the spectral amplitude of the noise.

The frequency spectrum shows up to two orders of magnitude reduction in noise at low frequencies and a smaller, but still significant, reduction at higher frequencies. Frames without diffraction signal contribute only to noise and can be treated as outliers and removed during analysis. In all the shot-to-shot analysis presented in the article, approximately 20% of the total collected frames were omitted.

**Figure 7:**
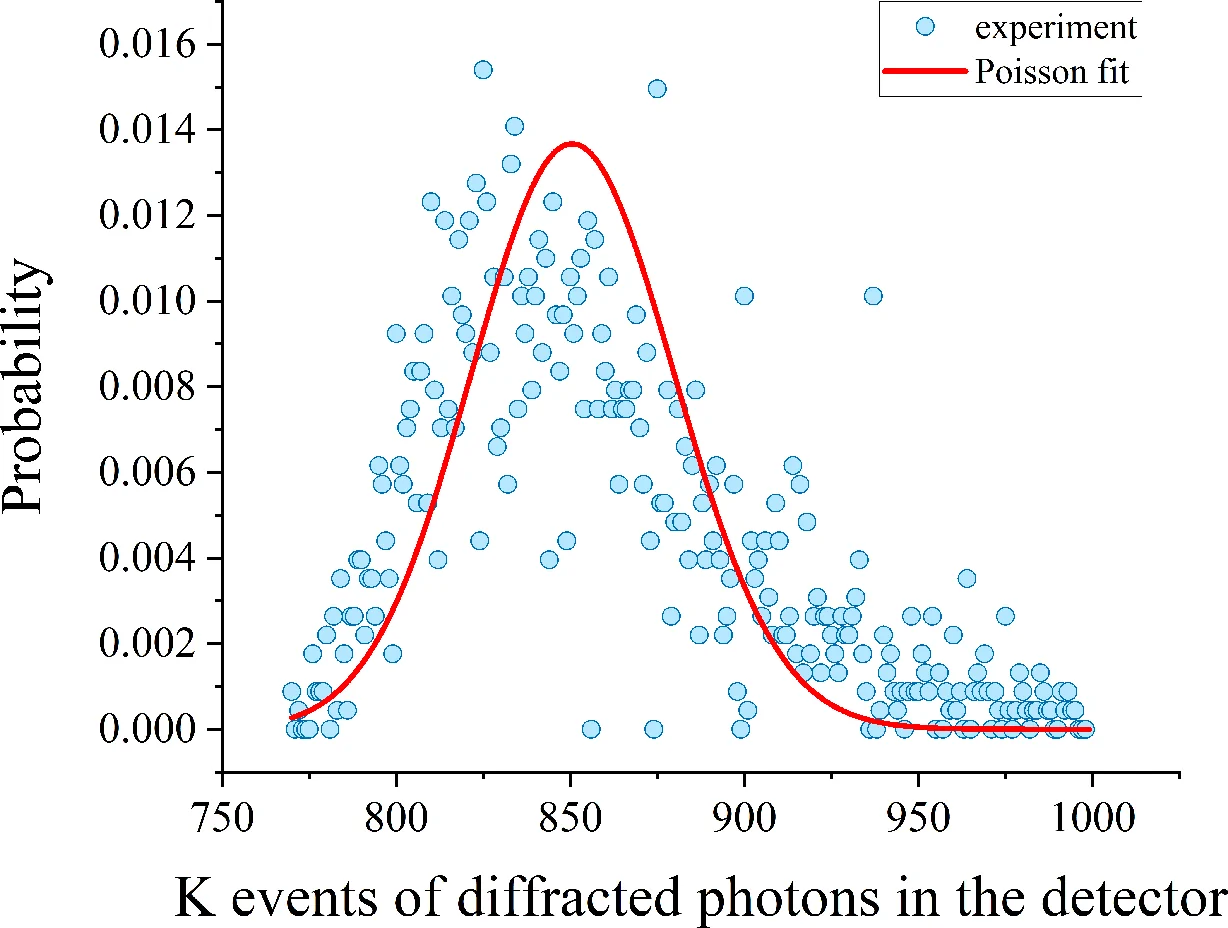
The probability distribution of shot-to-shot detected diffraction intensity from 4-phenylazophenol powder (in blue circles) and a Poisson fit shown by the red solid line. Here, K denotes the integrated intensity.

The probability distribution of the normalized integrated intensity for a diffraction peak at *q* = 1.45 Å^−1^ is shown in Fig. 7 ▸. The integrated shot-to-shot peak intensities were binned into 230 bins spanning the range 770–1000 events per 1000 shot and fitted with a Poisson distribution having a mean of 839. The goodness of fit yielded a χ^2^ value of 227.9, corresponding to a reduced χ^2^ of approximately 0.99, which indicates good agreement between the Poisson model and the experimental data. Thus, after normalization and removal of invalid frames, the distribution is well described by Poisson statistics, indicating shot-noise-limited behaviour.

**Figure 8:**
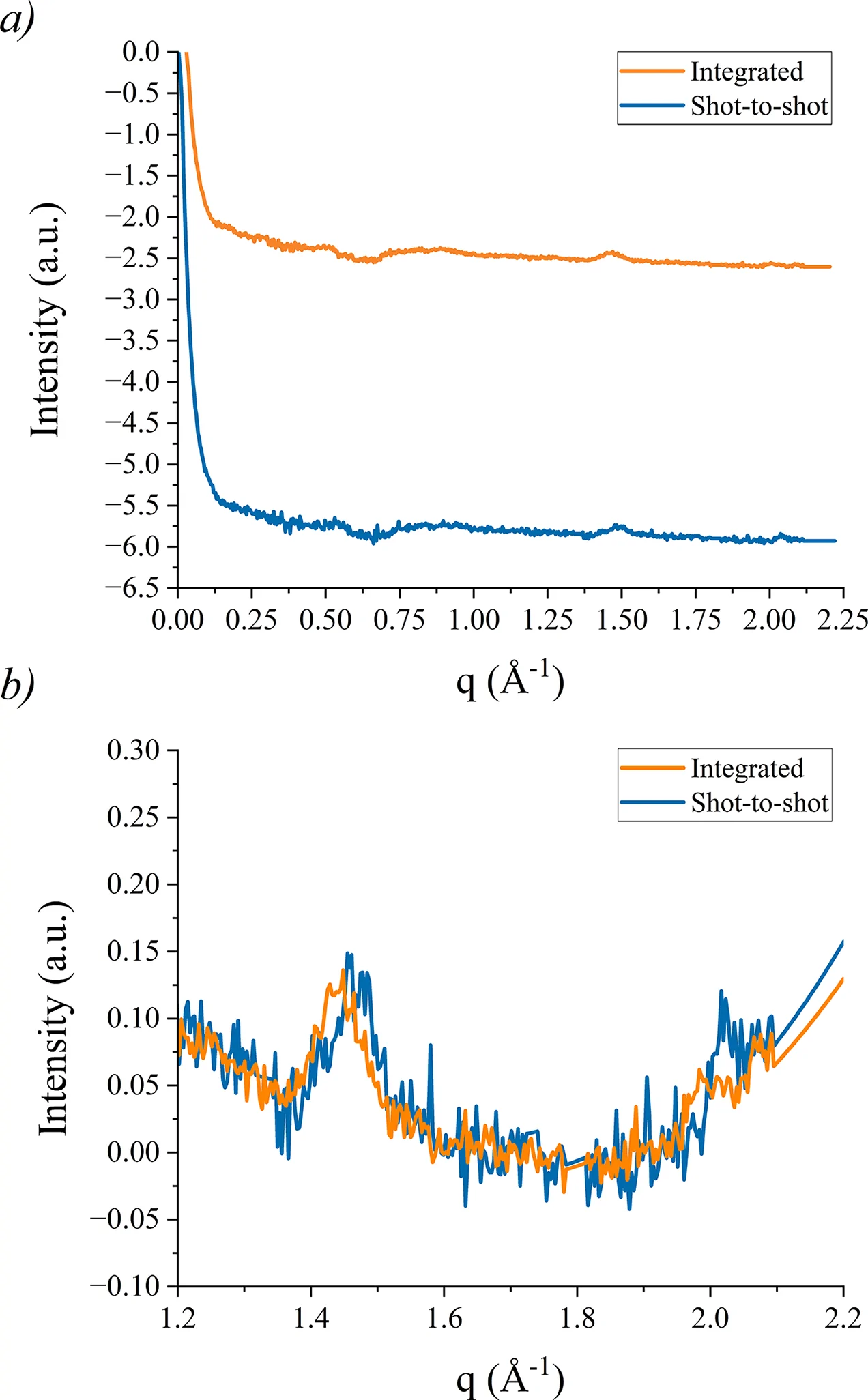
Comparison of PXRD profiles acquired in shot-to-shot mode and integrated mode (*a*) before baseline correction and (*b*) after baseline correction. A peak shift by 0.05 Å^−1^ is introduced to the shot-to-shot XRD in (*b*) to improve the visibility of the profiles in the two different conditions.

Finally, we compare azimuthally integrated PXRD patterns obtained using integrated PXS data and shot-to-shot PXS data [Fig. 8 ▸(*a*)]. For shot-to-shot analysis, frames not contributing to the expected diffraction signal were excluded. The baseline level differs significantly between integrated and shot-to-shot data, by up to several orders of magnitude. After baseline subtraction [Fig. 8 ▸(*b*)], the shot-to-shot data exhibit a slightly improved peak-to-valley ratio. Notably, a weak peak at ∼2.0 Å^−1^, barely visible in integrated data, becomes clearly distinguishable in shot-to-shot mode. Computing the peak-to-valley ratio for the peak at ∼2.0 Å^−1^ showed an enhancement of ∼2.4 times in the shot-to-shot data treatment.

## 5. Conclusion and outlook

In conclusion, we present shot-noise-limited PXRD from an organic crystalline powder using a laser-driven X-ray source. While demonstrated here for powder diffraction, the approach is directly applicable to single-crystal diffraction experiments as well. Application to an azobenzene derivative further suggests that transient structural dynamics during photoisomerization can be probed using such sources in the future. We demonstrate that shot-to-shot acquisition improves the signal-to-noise ratio by a factor of ∼2. This improvement has the potential to extend the detection of weak difference signals beyond what has previously been achieved with laser-driven X-ray sources (Zamponi *et al.*, 2012 ▸). Further improvements in shot-noise-limited PXRD may be achieved using machine learning approaches (Oppliger *et al.*, 2024 ▸), which could increase the range of detectable signals in such experiments. While shot-to-shot detection clearly provides significant advantages, it also introduces challenges in data handling, particularly when acquisition times extend to hours or days, as required for time-resolved diffraction studies. For detectors such as Jungfrau, these challenges could be mitigated by adopting an event-driven data format, in which only pixels with non-zero photon counts, together with their positions and frame indices, are stored. Such an approach would be similar to the readout scheme used in Timepix3 detectors (Poikela *et al.*, 2014 ▸). The current strategy relies on lossless image compression; however, for sparse datasets, compression ratios remain limited, and analysis still requires decompression and processing of full detector frames, which introduces unnecessary computational overhead. At low flux, additional improvements could be achieved through spectral filtering on individual detector frames (Martiel *et al.*, 2020 ▸) by rejecting pixels with intensities unlikely to originate from 8 keV photons, assuming that photon pile-up outside the direct beam region is negligible. Both event-based storage and spectral filtering are planned for future implementation within the Jungfraujoch system at TREX.

This work also highlights the unique capabilities of the TREX end station, which enables measurements using both continuous and femtosecond X-ray sources within the same setup. To our knowledge, such a combined system has not yet been reported. It provides the opportunity to perform X-ray pump/X-ray probe experiments on millisecond timescales, limited by the detector frame rate. With appropriate synchronization and the addition of choppers, the setup can be extended to microsecond or shorter timescales. With suitable choppers installed and introduction of the appropriate synchronization, the setup also facilitates X-ray pump/X-ray probe with microsecond or shorter timescales. Such capabilities are expected to be of significant interest for applications in radiochemistry and radiobiology (Verwohlt *et al.*, 2018 ▸). More broadly, the combination of optically driven and electronically driven X-ray sources, when properly synchronized, enables structural dynamics studies spanning timescales from femtoseconds to milliseconds and beyond. We believe that the developments presented here will be of broad interest to users of laser-driven X-ray sources aiming to probe challenging samples in transient structural studies.

## Supplementary Material

Supplementary figures. DOI: 10.1107/S1600576726009040/te5168sup1.pdf

## Data Availability

The data presented in the article are available on request to the corresponding author.
