# Supplementary material for: Shot-noise-limited X-ray powder diffraction from organic crystals with laser-driven femtosecond X-ray source
Source: J Appl Crystallogr. 2026 Sep 15;59(Pt 5):1707–13. doi: 10.1107/S1600576726009040 (PMC13636034; doi:10.1107/S1600576726009040)
Supplement: Supplementary file 1 [file j-59-01707-sup1.pdf]

JOURNAL OF
APPLIED
CRYSTALLOGRAPHY

Volume 59 (2026)

Supporting information for article:

Shot-noise Limited X-ray Powder Diffraction from Organic Crystals with Laser-driven Femtosecond X-ray Source

Iuliia Baranova, Yelyzaveta Pulnovaa, Roya Garayeva, Yadu Ram Panthi, Gabriel Žoldák, Jaroslav Nejdl, Borislav Angelov, Filip Leonarski, Jakob Andreasson and Krishna Khakurel

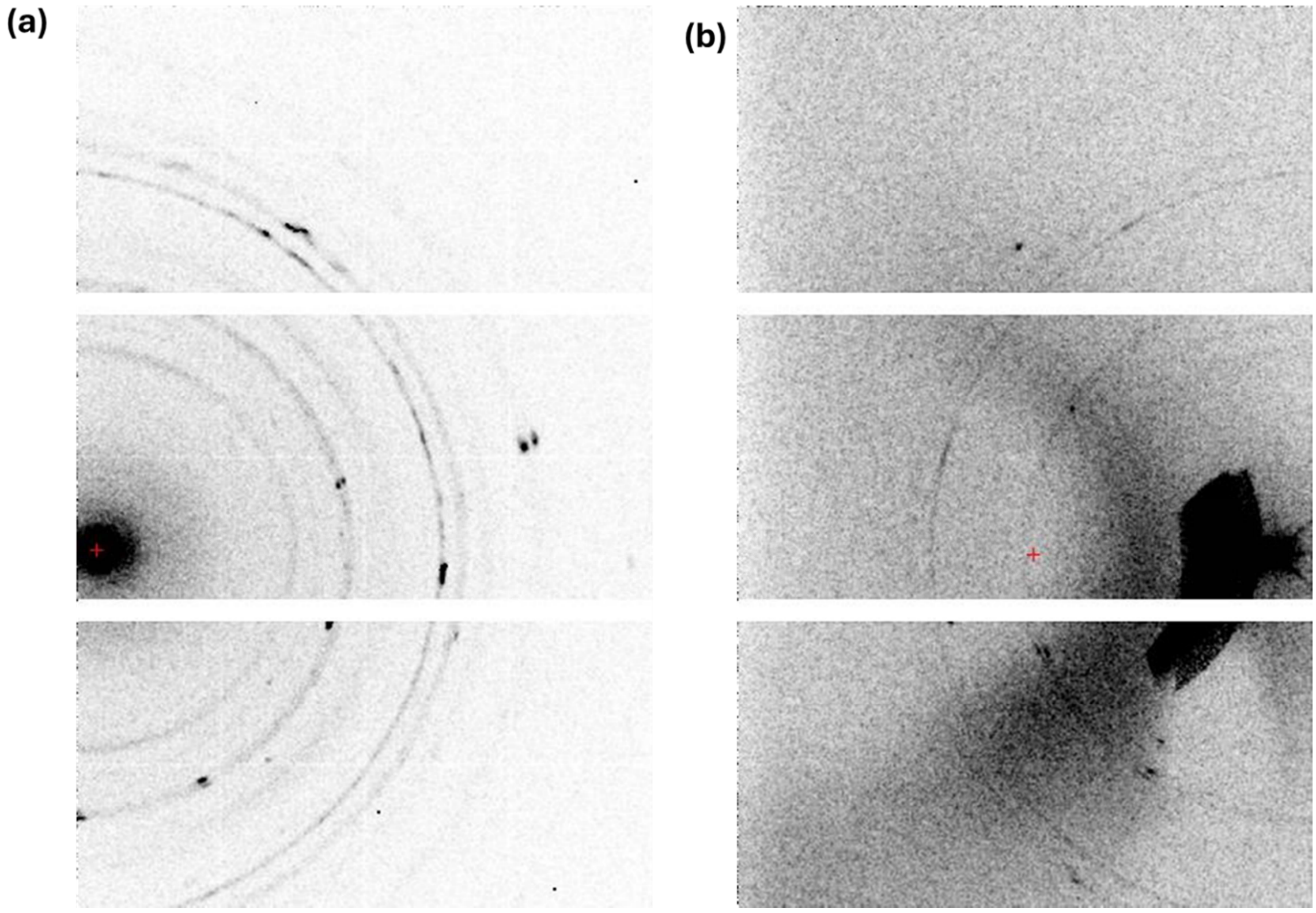

Supplementary Figure 1. Two-dimensional X-ray powder diffraction from 4-Phenylazophenol (a) collected with a continuous X-ray tube source (b) collected with the laser-driven plasma X-ray source.

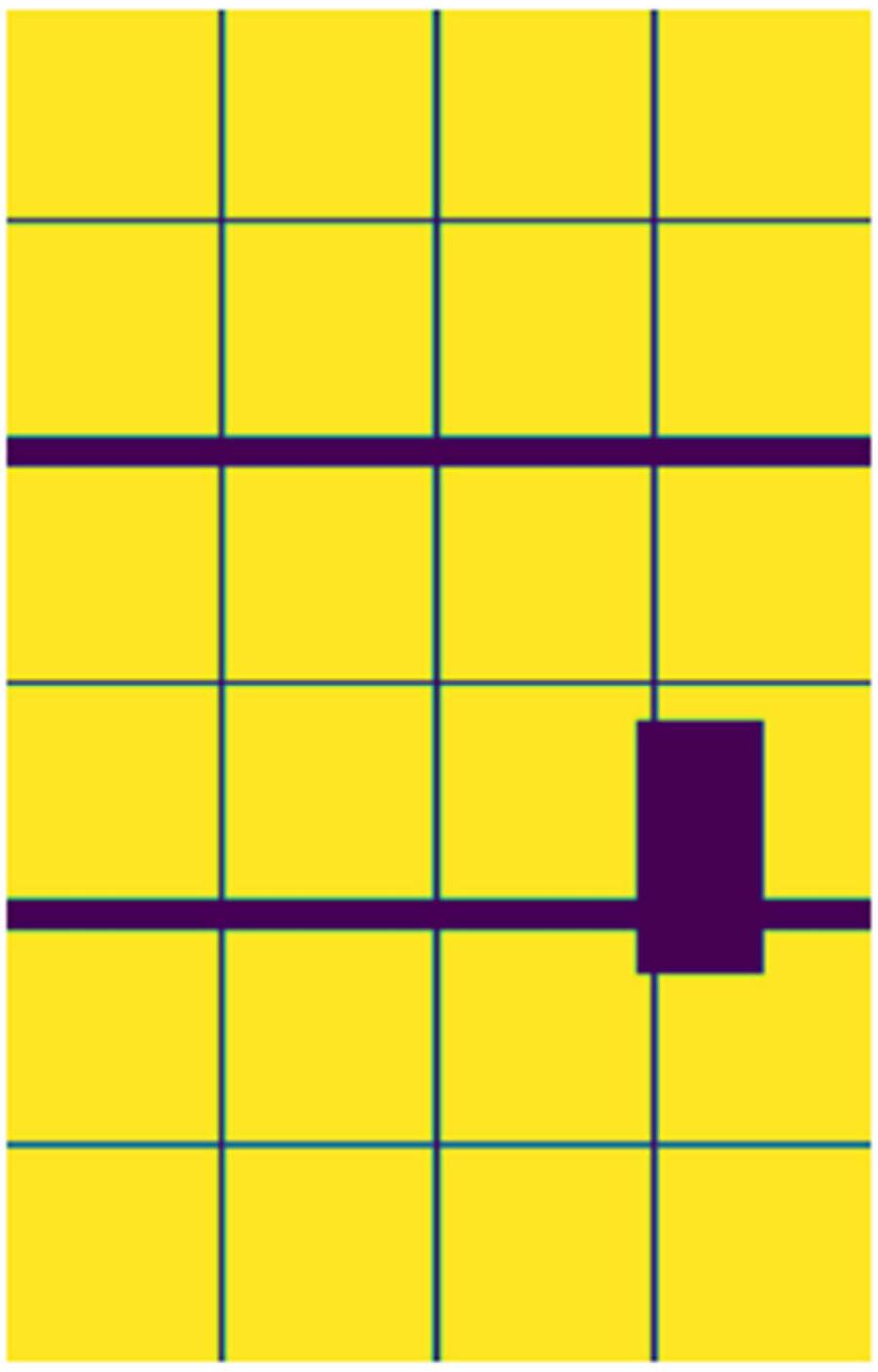

Supplementary Figure 2. Binary mask used to remove the dead zones of the detector and the stray scattering.

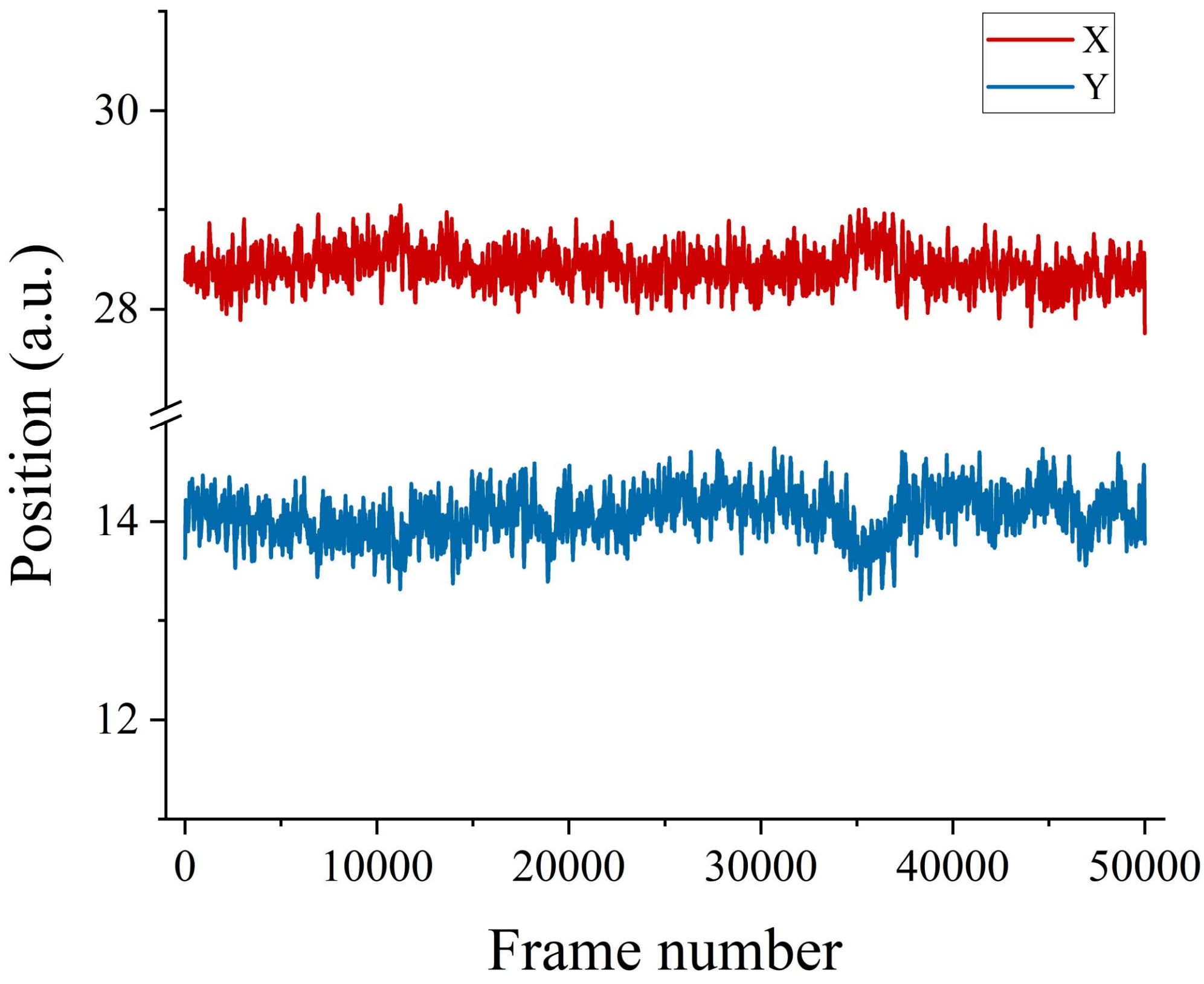

Supplementary Figure 3. Change in the position of the beam center along X and Y axes from shot to shot over 50 s.
